# Study of microRNA expression in *Salmonella* Typhimurium-infected porcine ileum reveals miR-194a-5p as an important regulator of the TLR4-mediated inflammatory response

**DOI:** 10.1186/s13567-022-01056-7

**Published:** 2022-05-21

**Authors:** Juber Herrera-Uribe, Sara Zaldívar-López, Carmen Aguilar, Carmen Entrenas-García, Rocío Bautista, M. Gonzalo Claros, Juan J. Garrido

**Affiliations:** 1 Immunogenomics and Molecular Pathogenesis Group, Department of Genetics, Faculty of Veterinary Medicine, University of Córdoba, Córdoba, Spain; 2 Maimónides Biomedical Research Institute of Córdoba (IMIBIC), Research Group GA-14, Córdoba, Spain; 3 Andalusian Platform of Bioinformatics-SCBI, University of Málaga, Málaga, Spain; 4 Department of Molecular Biology and Biochemistry, University of Málaga, Málaga, Spain; 5 Viral Immunology Group, School of Biochemistry and Immunology, Trinity College Dublin, Trinity Biomedical Sciences Institute, Dublin, Ireland; 6 Institute for Molecular Infection Biology (IMIB), University of Würzburg, Würzburg, Germany

**Keywords:** Salmonellosis, microRNAs, inflammation, Toll-like receptor, ileum, immunity, infection, pig, miRNA-seq, CRISPR-Cas9

## Abstract

Infection with *Salmonella* Typhimurium (*S*. Typhimurium) is a common cause of food-borne zoonosis leading to acute gastroenteritis in humans and pigs, causing economic losses to producers and farmers, and generating a food security risk. In a previous study, we demonstrated that *S*. Typhimurium infection produces a severe transcriptional activation of inflammatory processes in ileum. However, little is known regarding how microRNAs regulate this response during infection. Here, small RNA sequencing was used to identify 28 miRNAs differentially expressed (DE) in ileum of *S*. Typhimurium-infected pigs, which potentially regulate 14 target genes involved in immune system processes such as regulation of cytokine production, monocyte chemotaxis, or cellular response to interferon gamma. Using in vitro functional and gain/loss of function (mimics/CRISPR-Cas system) approaches, we show that porcine miR-194a-5p (homologous to human miR-194-5p) regulates *TLR4* gene expression, an important molecule involved in pathogen virulence, recognition and activation of innate immunity in *Salmonella* infection.

**Supplementary Information:**

The online version contains supplementary material available at 10.1186/s13567-022-01056-7.

## Introduction

Salmonellosis ranks second within the most notified zoonosis in Europe, with 42.5% of the cases needing hospitalization. *Salmonella* Typhimurium (*S*. Typhimurium) is the second most reported serovar, and the most commonly found pathogen in pork meat (especially in Spain, which contributed with 30.4% of the European cases). Although many control programs are currently established in farms, the number of cases in Europe has not decreased in the last 5 years, therefore better control measures need to be implemented, as 14.3% of pig carcasses in Spain are *Salmonella*-positive [1].

Following ingestion, *Salmonella* reaches the small intestine, where it adheres to the intestinal mucosa as the first step in the pathogenesis of infection. After invasion, pathogen associated molecular patterns (PAMPs) are recognized by pattern-recognition receptors (PRRs) from intestinal epithelial cells such as membrane bound Toll-like receptors (TLRs), which stimulate the host immune response [2]. TLR4 recognizes lipopolysaccharide (LPS) in the cell wall of Gram negative bacteria, activating signaling cascades that regulate the expression of pro-inflammatory cytokines and chemokines, leading to the recruitment of macrophages, lymphocytes, and polymorphonuclear leukocytes [3]. A vast innate immune response is elicited by the host at the site of infection and, although counterintuitive, it has been shown that the intestinal section with the greatest inflammatory changes (i.e. ileum) is also the most colonized by *Salmonella*, suggesting that this pathogen benefits from inflammation to further colonize the tissues [4]. Further research regarding the nature of the host–pathogen interaction at the infection site is needed to better understand *Salmonella* infection and its regulation. Previous studies have shown that microRNAs (miRNAs), which are conserved small non-coding post-transcriptional regulators of gene expression [5], modulate the development and function of immune cells and can have pro- or anti-inflammatory effects during bacterial infections [6, 7]. The regulation of the transcriptional response by miRNAs in *Salmonella* infection is still mostly unclear, but several miRNAs such as let-7i or miR-15 have been shown to modulate certain host immune functions [8, 9]. In a previous study, we described the transcriptomic response in the porcine ileum 2 days after infection with *S*. Typhimurium, as well as the expression of a set of miRNAs in this tissue, using microarray analysis [4]. However, the recent development of powerful miRNA-specific sequencing techniques as small RNA-seq prompted us to perform a deeper evaluation and quantification of miRNA expression in this experimental setting. The purpose of this study was to elucidate the role of miRNAs in the regulation of the inflammatory processes that are elicited after *Salmonella* infection in ileum. Additionally, we identified key miRNAs that influence the host response to this pathogen.

## Materials and methods

### Experimental infection and sample processing

The experimental infection design was previously described elsewhere [10]. Briefly, eight female crossbred weaned piglets (commercial hybrids of Landrace × Large White × Pietrain), confirmed to be fecal-negative for *Salmonella*, were used in this study. Four piglets per group were randomly allocated to control and infected groups. Control piglets were necropsied 2 h before the experimental infection, whereas the 4 remaining piglets were challenged orally with 10^8^ colony forming units (cfu) of a natural isolate of *S.* Typhimurium phage type DT104 [11], and necropsied 2 days post-infection (2 dpi). Ileum samples (segments approximately 10 cm long) were collected and immediately frozen in liquid nitrogen.

For intestinal mucosa isolation and RNA purification, tissue samples stored at −80 °C were temperature-transitioned with RNAlater^®^-ICE (Ambion Inc, Austin, TX, USA) and cut into 2 cm pieces. Intestinal mucosa was scraped from the intestinal luminal surface with a sterile razor, and immediately disrupted and homogenized in lysis buffer (Ambion Inc, Austin, TX, USA) using a rotor–stator homogenizer. RNA extraction was performed using mirVana miRNA isolation kit (Ambion Inc., Austin, TX, USA). Eluted RNA was treated with DNase using TURBO DNA-free^™^ Kit (Ambion Inc., Austin, TX, USA) to eliminate traces of DNA. RNA integrity was assessed in the Agilent Bioanalyzer 2100 (Agilent Technologies, Palo Alto, CA, USA), and only samples with RNA integrity numbers (RIN) ≥ 7 were used for sequencing and further analysis.

### Small RNA library preparation, sequencing and data analysis

Four samples (2 controls and 2 *S*. Typhimurium infected) were used for small RNA sequencing (small RNA-seq). Sample quality control, library creation and sequencing were performed at the Functional Genomics Core at the Institute for Research in Biomedicine in Barcelona (IRB Barcelona). Five hundred nanograms of total RNA per sample were used for library preparation (NEBNext^®^ Multiplex Small RNA Library Prep Set for Illumina, New England Biolabs Inc, Ipswich, MA, USA). Libraries were quantified with Qubit dsDNA HS assay (Thermo Fisher Scientific Inc., Waltham, MA, USA) and quality was assessed using an Agilent Bioanalyzer 2100 (Agilent Technologies, Palo Alto, CA, USA). Then, single-end next-generation small RNA sequencing of 50 nucleotides-long reads was performed using HiSeq2000 sequencing platform (Illumina Inc., San Diego, CA, USA).

Data analysis was performed at the Andalusian Platform of Bioinformatics at the University of Malaga. Raw reads were pre-processed using the in-house developed customizable pipeline SeqTrimNext [12]. Contaminants, sequencing adapters, short (<17 nucleotides) and bad quality reads (Phred score <20) were removed, so only high-quality sequences were used for further analyses. The miRNA database used was miRbase (release 22.1). For the analysis of deep sequencing data, we used the CAP-miRSeq pipeline (alignment, miRNA detection, quantification, and differential expression analysis between control and infected group) [13]. This pipeline includes an alignment to the pig genome (Sscrofa11.1) with bowtie1 [14] and miRNA identification with miRDeep2 algorithms [15]. CAP-miRSeq also implements edgeR for determination of differential expression between control and infected samples, which uses empirical Bayes estimation and exact test based on the negative binomial distribution [16]. miRNAs were considered to be differentially expressed (DE) if false discovery rate (FDR) corrected *p*-value was < 0.05 (Benjamini–Hochberg method), and fold change (FC) was ≥2 (absolute value).

### Quantitative real-time PCR (qPCR) and analysis

Eight samples (4 controls and 4 *S*. Typhimurium infected) were used for miRNA qPCR and gene validation. Selected miRNAs DE in the sequencing analyses and target genes were validated as previously reported [17] using specific primers (Additional file 1). Briefly, to conduct miRNA qPCR analysis, 100 ng of total RNA per animal were reverse transcribed to cDNA, which was 1:8 diluted and added to a 10 µL PCR reaction mix containing 2 µL of 5× PyroTaq EvaGreen qPCR Mix Plus with ROX (Cultek Molecular Bioline, Madrid, Spain), and 10 µM of each primer. Cycling conditions were 10 min at 95 °C followed by 40 cycles of 5 s at 95 °C, and 60 s at 60 °C; a final melting curve analysis was performed (60–99 °C).

Likewise, miRNA-predicted target genes were analyzed by qPCR, for which RNA samples (1 µg) were reverse-transcribed using qScript^™^ cDNA synthesis kit (Quanta Biosciences Inc.), following manufacturer’s instructions. The final 15 µL PCR reaction included 2 μL of 1:10 diluted cDNA as template, 3 µL of 5× PyroTaq EvaGreen qPCR Mix Plus with ROX (Cultek Molecular Bioline, Madrid, Spain), and transcript-specific forward and reverse primers at a 10 μM final concentration. Real-time PCR was carried out in a QuantStudio 12K Flex system (Applied Biosystems, Waltham, MA, USA) under the following conditions: 15 min at 95 °C followed by 35 cycles of 30 s at 94 °C, 30 s at 57 °C and 45 s at 72 °C. Melting curve analyses were performed at the end to ensure the specificity of each PCR product.

Expression results were calculated using GenEx6 Pro software (MultiD, Göteborg, Sweden), based on the Cq values obtained. Based on the literature [18, 19] and after qPCR analysis using GeNorm GenEx6 tool, the most stable miRNAs (miR-26a, let-7a, miR-103, miR-17-5p and miR-16-5p) and genes (*B2M*, *CYPA* and *RPL4*) were selected as reference to normalize expression. Relative gene expression was measured in control and infected pigs, resulting in expression ratios calculated according to the 2^−ΔΔCt^ method [20]. Statistical differences in expression among groups were assessed using Student’s *t* test (GraphPad Prism 6, GraphPad Software Inc, La Jolla, CA, USA). Statistical significance was set at *P* < 0.05. Additionally, a Pearson correlation analysis was performed between small RNA-Seq and qPCR results to validate the sequencing results. Heatmaps were created using the gplots package (v3.0.1) within the RStudio software (v. 1.0.143).

### miRNA-target gene selection, integrative and functional analysis

miRNA target genes were selected using the miRNA target database miRTarbase (release 6.0) and TargetScan (release 7.0) [11, 21]. In order to increase the confidence of the findings, we selected only output targets with strong evidence in their validation (performed by reporter assay, Western blot or qPCR), and those miRNA target genes with highly conserved seed regions. We compared all miRNA targets predicted from DE miRNAs in ileum at 2 dpi with DE mRNAs from our previously published gene expression study from the same samples [4]. Selection of miRNA targets was performed based on the nature of miRNA regulation, where upregulated miRNAs from our DE dataset were paired with down- or up-regulated mRNA from our DE gene dataset [4], and downregulated miRNAs were paired with upregulated mRNA from the same gene expression study. Functional enrichment analysis of selected target genes was performed using ClueGO [22], a Cytoscape open-source Java tool plug-in [23]. Terms were classified in a functional group (GO term fusion), and the name of the functional groups was given by the statistical significance of the leading term. Statistical significance was set as follows: Benjamini–Hochberg corrected *P* < 0.05, κ score = 0.5 and at least 3 genes per term.

### In vitro miRNA functional assays and target validation

microRNA mimic hsa-miR-194-5p (cat #: C-300642-03-0005, homologous to ssc-miR-194a-5p), and mimic negative control (cat#: CN-001000-01-05) were obtained from Dharmacon (Horizon Discovery Ltd., UK). Mimic miR-194-5p and negative control were transfected into intestinal porcine enterocytes cell line (IPEC-J2 cells) using Viromer Blue reagent (Lipocalyx GmbH, Germany) following the reverse transfection protocol recommended by the manufacturer, at a final miRNA concentration of 50 nM. Briefly, 7.5 × 10^4^ cells (per well) were resuspended in DMEM/F-12 (Life Technologies, Waltham, MA, USA) medium supplemented with 5% fetal bovine serum (Life Technologies, Waltham, MA, USA), mixed with transfection solution, and seeded on 24-well plates (Thermo Fisher Scientific, Waltham. MA, USA). Transfected cells were incubated at 37 °C in a 5% CO_2_ humidified atmosphere for 48 h, and then infected with the *S*. Typhimurium phage type DT104 [11] previously used in the in vivo experimental infection (OD_600_ = 0.8, MOI 1:25). After 1 h of infection, the medium was replaced with fresh medium containing gentamicin (100 µg/mL) to kill extracellular bacteria and incubated for 2 h. Cells were lysed for RNA isolation with mirVana miRNA isolation kit (Ambion Inc., Austin, TX, USA).

### miRNA-target validation by luciferase assay

miRNA recognition elements (MREs) were predicted with TargetScan and RNAhybrid [21, 24]. The hybridization energy required for the formation of the miRNA-MRE duplex was calculated by uploading to RNAhybrid the sequence of 3'UTR segments containing the MREs and their respective predicted miRNA. Only the duplexes with favorable hybridization energy of ≥ −15 kcal/mol were chosen as potential MREs. The 3′UTR of TLR4, predicted to be targeted by miR-194-5p, was amplified by PCR and cloned into the firefly luciferase in the psiCHECK2 vector. Primer sequences and restriction enzymes used for cloning the 3′UTR of porcine genes are shown in Additional file 1. *E. coli* cells transformed with a recombinant miRNA target expression vector (psiCHECK2) were grown overnight in the appropriated volume of LB medium with ampicillin (100 μg/mL). Plasmid DNA was isolated using the JetStar 2.0 Plasmid Purification Kit system (Genomed GmbH, Löhne, Germany) according to the manufacturer’s protocol.

Mimic miR-194-5p and negative control were transfected individually into Chinese Hamster Ovary cells (CHO) at a final miRNA concentration of 75 nM. Cells were cultured in RPMI medium (Biowest, Nuaillé, France) supplemented with 10% of heat-inactivated fetal calf serum (Gibco, Life Technologies, Waltham, MA, USA) and 2 mM l-glutamine (Biowest, Nuaillé, France), at 37 °C and 5% CO_2_. After incubation of the transfection mix, 2 × 10^4^ cells (per well) were resuspended in RPMI medium with 10% of heat-inactivated fetal calf serum (Gibco, Life Technologies, Waltham, MA, USA) and 2 mM l-glutamine (Biowest, Nuaillé, France), mixed with transfection solution and seeded on 96-well plates (Thermo Fisher Scientific, Waltham, MA, USA). Transfected cells were incubated (37 °C in 5% CO_2_ humidified atmosphere) and after 24 h cells were co-transfected with 250 ng of the psiCHECK2 vector constructions, using Lipofectamine 3000 transfection kit (Invitrogen, Life Technologies, Waltham, MA, USA). After 48 h of incubation, cells were washed twice with PBS and lysed with 50 µL of 1× passive lysis buffer (Promega, Madison, WI, USA). An aliquot of 20 µL was assayed for firefly and renilla luciferase activity using the dual luciferase reporter assay system (Promega, Madison, WI, USA) according to the manufacturer’s protocol. Luciferase activity values were obtained by a Varioskan Lux luminometer (ThermoFisher Scientific, Waltham, MA, USA). Control experiments were performed for each putative target, including plasmids that did not contain the 3′UTR fragment and negative controls of miRNA mimic. Statistical differences in expression values among groups were assessed using a Student’s *t-*test (GraphPad Prism 6, GraphPad Software Inc, La Jolla, CA, USA), with statistical significance set at *P* < 0.05.

### Inhibition of miR-194a-5p expression via CRISPR/Cas9 system

Plasmid pSpCas9(BB)-2A-Puro (PX459) was purchased from the Addgene plasmid repository (Cambridge, MA, USA). miR-194a-5p guides (gRNAs) were designed using the MIT CRISPR Design Tool [25]. The gRNAs targeting porcine miR-194a-5p genomic sequences were cloned into the plasmid pSpCas9(BB)-2A-Puro following the manufacturer’s instructions, and verified by DNA sequencing. Two hundred and fifty nanogram per microliter of each gRNA-containing plasmid were transfected with Viromer Yellow (Lipocalyx GmbH, Germany) into IPEC-J2 cells using a reverse transfection protocol. Puromycin treatment (2 μg/mL for 24 h; Life Technologies, Waltham, MA, USA) was used for selection, and then transfected cells were isolated through serial dilutions in the culture medium. Clones obtained by this method were characterized by PCR and DNA sequencing.

### Gentamicin protection assay

Clones of CRISPR/Cas9, IPEC-J2 cells transfected with miR-194-5p mimic and controls were infected with *S*. Typhimurium as mentioned above. After infection, monolayers were washed twice with PBS containing gentamicin (100 μg/mL), then the media was replaced with fresh media containing gentamicin (100 μg/mL) to kill extracellular bacteria. After 2 h of incubation, monolayers were washed twice with PBS and lysed with 1% Triton X-100 solution. Lysates were vigorously vortexed for 1 min, diluted and plated in TSA medium (Trypticase soy agar). Invasiveness was calculated by counting the colony-forming units (c.f.u.). The experiments were conducted in triplicate on three different days. Statistical differences were assessed using Student’s *t*-test (GraphPad Prism 6, GraphPad Software Inc., La Jolla, CA, USA) and differences were set at *P* < 0.05.

### Detection and quantification of intracellular *S*. Typhimurium by TaqMan qPCR

TaqMan qPCR assay previously described by Martins et al. [26], was used to quantify concentrations of *S*. Typhimurium in IPEC-J2 cells and CRISPR/Cas 9 clones. *S*. Typhimurium standard curve was performed using DNA from a pure broth of the *Salmonella* strain used in this study. *Salmonella* DNA was isolated using DNeasy Blood & tissue kit (Qiagen, Valencia, CA, USA). Subsequently, known concentrations of 1.0 × 10^5^, 5.0 × 10^4^, 1.0 × 10^4^, 5.0 × 10^3^, 1.0 × 10^3^, 5.0 × 10^2^, 1.0 × 10^2^, 5.0 × 10^1^, and 0 genome equivalents (GE) per 1 μL of DNA were used to build the reference standard curve, in which 1 GE of *S*. Typhimurium corresponded to 5.46904 fg of DNA. A 19-mer forward primer (5′-GCGCACCTCAACATCTTTC-3′), a 22-mer reverse primer (5′-GGTCAAATAACCCACGTTCA-3′), and a fluorogenic probe (FAM ATCATCGTCGACATGC MGB/NFQ) were used in the quantification assays. Twenty-five microliters of PCR reactions contained 12.5 μL IQ Supermix 2× (Biorad, Madrid, Spain), 0.4 μM of each primer, 0.2 μM probe, 1 μM MgCl_2_, 200 ng DNA, and 10 μL UHQ water. PCR amplifications were performed on an iQ5 Thermo Cycler (Biorad, Madrid, Spain) under the following conditions: 95 °C for 10 min and 50 cycles of 95 °C for 15 s and 60 °C for 1 min. Statistical differences were assessed using Student’s *t*-test (GraphPad Prism 6, GraphPad Software Inc., La Jolla, CA, USA) and differences were set at *P* < 0.05.

## Results

### *S*. Typhimurium infection downregulates miRNA expression in porcine ileal mucosa

Sequencing yielded about 7.48 (SD 0.54) million raw reads per sample which, after removing the adapters, filtering the quality of the sequence (Phred score > 20) and length of the reads, resulted in about 2.17 (SD 0.51) millions of clean output reads. The read length distribution observed in our samples was consistent with profiles generated in other studies [27], with the highest number of reads within the 19–25 nucleotides range.

We first characterized the miRNA expression profile in control and infected intestinal samples. miRNAs with at least one mapped read in each library (control or infected) were selected for determining the miRNA expression profile in the ileum. Analysis revealed that 312 annotated miRNAs were expressed in ileum from infected pigs, while 311 miRNAs were expressed in ileal mucosa from non-infected control animals. The 25 most abundantly expressed miRNAs in porcine ileum (both control and infected groups) are shown in Additional file 2. The most abundantly expressed miRNA was miR-21; miR-143-3p, miR-192, miR-26a, miR-215 and mir-148a-3p were also highly expressed in the porcine ileum.

**Figure 1:**
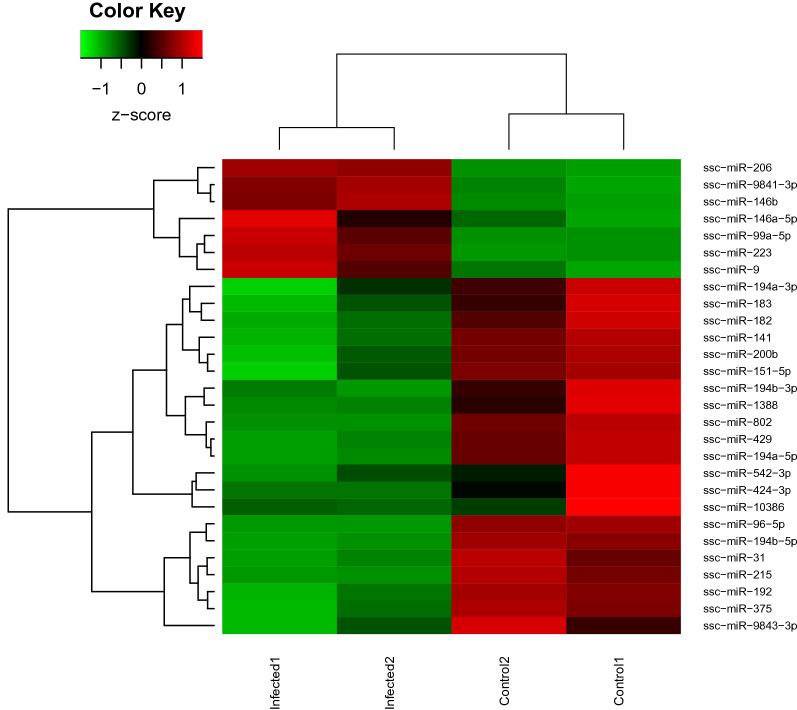
**Differentially expressed miRNAs in control and infected samples.** Twenty-eight miRNAs were found DE (*p*-value < 0.05 and a FC ≥ 2) in *Salmonella* Typhimurium infected ileal samples. Heatmap shows overexpression (red) and repression (green) of porcine miRNAs.

**Figure 2:**
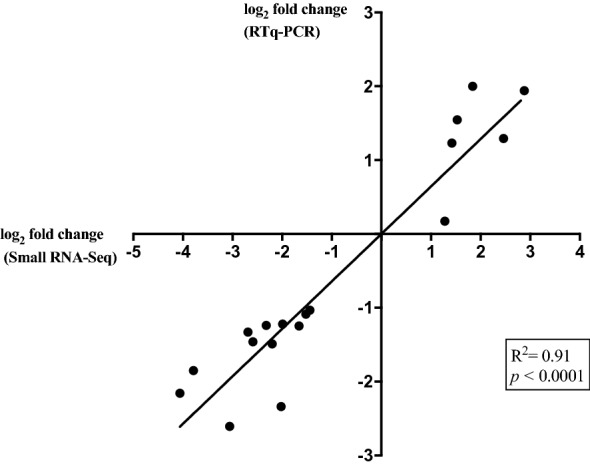
**qPCR validation of sequencing results.** Pearson correlation analysis of FC values of DE miRNAs between qPCR and RNA-seq analysis, values showed a highly significant and strong positive correlation.

Compared with the uninfected group, a total of 28 miRNAs were found DE in *Salmonella*-infected samples. Of these, 21 were significantly down-regulated, while 7 were up-regulated (Figure 1, Additional file 3). Down-regulation of the miR-200 family (miR-200b and miR-141), miR-215 and miR-192 as well as up-regulation of miR-146a, miR-146b and miR-223 were detected, among others. Interestingly, we found that all four mature forms of miR-194 (ssc-miR-194a-5p, ssc-miR-194a-3p, ssc-miR-194b-5p and ssc-miR-194b-3p,) were highly downregulated. To validate the accuracy of the sequencing data and bioinformatic analysis, we conducted qPCR of selected DE miRNAs. qPCR analysis confirmed the differential expression of 15 statistically significant up- and down-regulated miRNAs after *S.* Typhimurium infection, and results were in agreement with the deep sequencing results (Pearson correlation coefficient > 0.9, Figure 2).

### Integrative analysis of miRNA and gene expression data in porcine ileum after infection with *S*. Typhimurium

**Figure 3:**
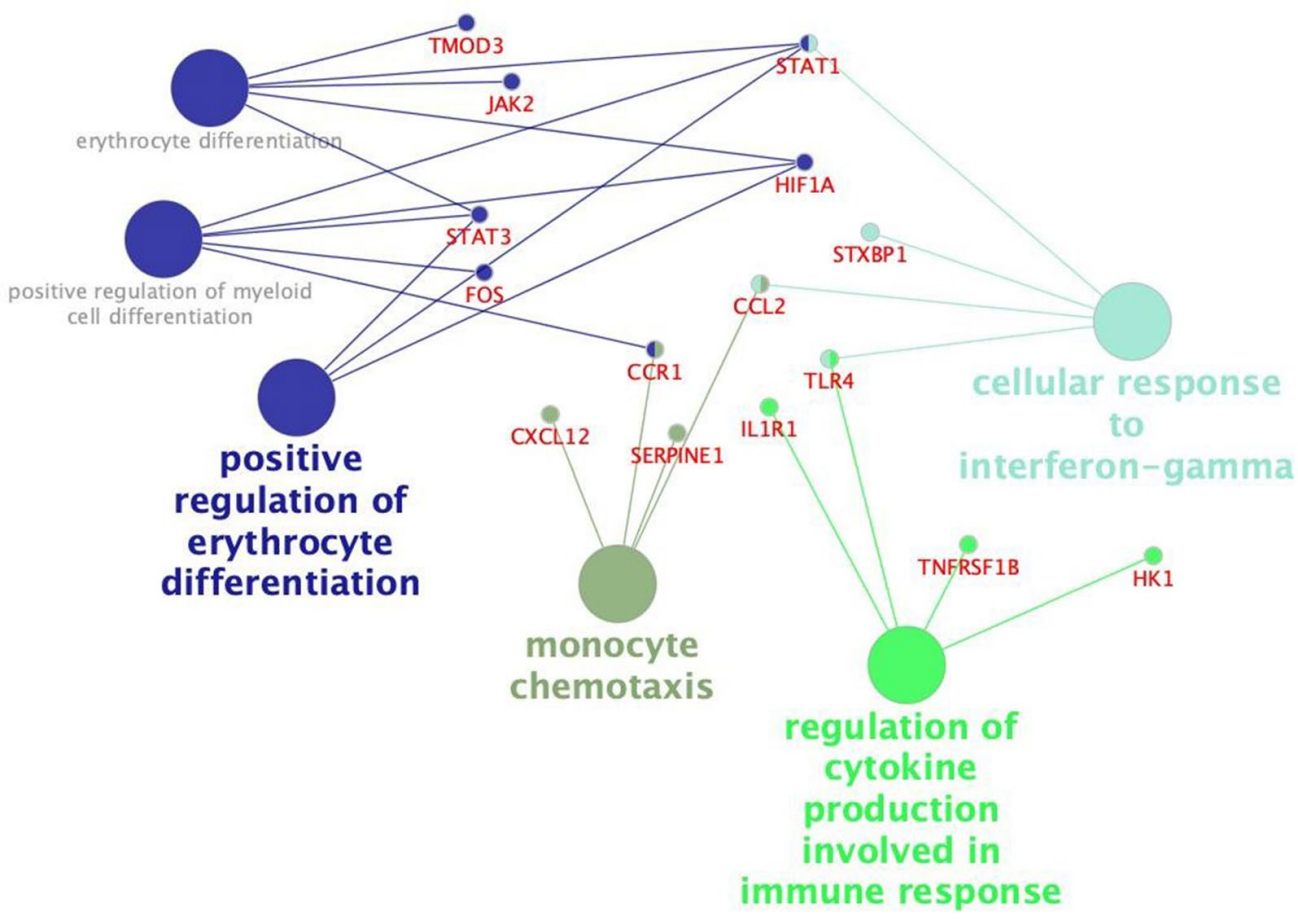
**Biological functions where miRNA predicted targets are involved.** This Figure shows the immune functions affected by DE miRNA target genes in *Salmonella* Typhimurium infection at 2 dpi. All the represented pathways are highly significant following a Benjamini–Hochberg correction.

To better understand the biological function of the 28 DE miRNAs in the ileal mucosa of *Salmonella*-infected pigs, target genes were predicted using TargetScan 7.0 and miRTarbase 6.0 databases. According to this, each microRNA would regulate hundreds of genes since computational tools predict microRNA targets by evolutionarily conserved microRNA binding sites. To focus on the most biologically relevant target genes, obtained predictive data was compared with a gene expression dataset from a previous study [4]. In this integrative analysis, we found that 193 DE genes in the ileum were likely regulated by DE miRNAs from this study (Additional file 4). Based on the nature of miRNA regulation, miRNA-mRNA pairing was performed as follows: 6 upregulated miRNAs were paired with 130 down- or up-regulated mRNA, and 16 downregulated miRNAs were paired with 233 upregulated mRNAs. Gene ontology analysis focused on immune response revealed biological functions where target genes were involved (Figure 3, Additional file 5). These functions include monocyte chemotaxis, regulation of cytokine production and cellular response to interferon gamma. DE miRNAs such as miR-223, miR-146, miR-802 and miR-542 were predicted to regulate DE genes such as *TLR4*, *STAT1/3*, *IL1R1* or *CCL2* (Additional file 4). Although *TLR4* was not among the initially predicted miR-194 target genes, reports have demonstrated that this miRNA regulates the TLR4 pathway [28, 29]. Given the importance of this signaling route in *Salmonella* infection, and its previously shown over-activation in *S*. Typhimurium-infected porcine ileum [4], we hypothesized that miR-194 could be directly regulating the TLR4 signaling pathway.

### Effect of miR-194 overexpression on TLR4 and downstream genes during *S*. Typhimurium infection

**Figure 4:**
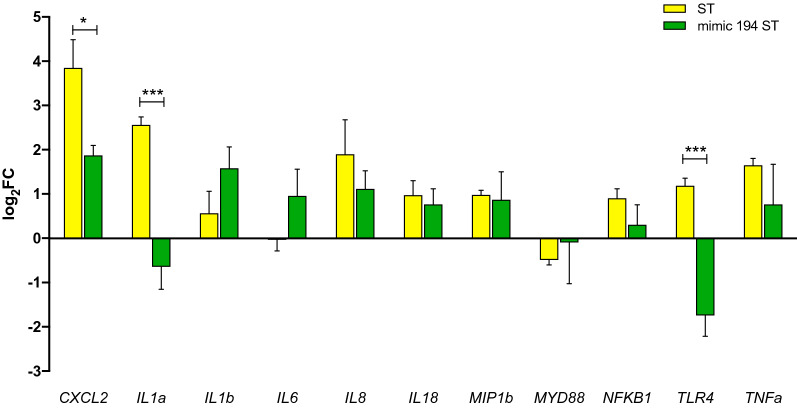
**Expression of potential target genes from the TLR4 pathway in** ***S.*** **Typhimurium infected IPEC-J2 cells after miRNA mimic transfection.**Gene expression of miR-194 in IPEC-J2 cells infected with *S*. Typhimurium (2 hpi, yellow bars) and IPEC-J2 cells transfected with miR-194 mimic and infected with *S*. Typhimurium (dark green bars). Bars represent mean log2 fold change compared to their respective controls, and standard error of the mean (SEM).

To elucidate the effect of miR-194, the expression of potential inflammation-related target genes was evaluated following miRNA mimics-mediated overexpression in IPEC-J2 cells infected with *S*. Typhimurium. Given that all mature forms of miR-194 showed the same expression tendency, we selected ssc-miR-194a-5p (homologous to hsa-miR-194-5p) for further studies based on reported expression on miRBase and abundancy of reads and *p*-value on our own sequencing results. We evaluated genes involved in the TLR4 pathway [30] and, similarly to the in vivo experiment (infected ileum mucosa), we found increased expression of *IL1α*, *IL1β*, *TLR4*, *IL8*, *IL18*, *NFκB1*, and *MIP1β* in *Salmonella*-infected IPEC-J2 cells compared to non-infected controls (Figure 4). When we increased the expression of miR-194a-5p using mimic transfection, overexpression of *IL1α* and *CXCL2* was significantly inhibited (*p* < 0.05) in transfected cells. Surprisingly, the expression of *TLR4* was also significantly inhibited. Genes such as *IL1β*, *IL6*, *IL8*, *TNFα*, *MYD88* and *NFκB1* did not show significant changes after transfection with miR-194-5p mimic.

### Luciferase reporter assay and CRISPR/Cas9 system revealed TLR4 to be a target gene of miR-194-5p

**Figure 5:**
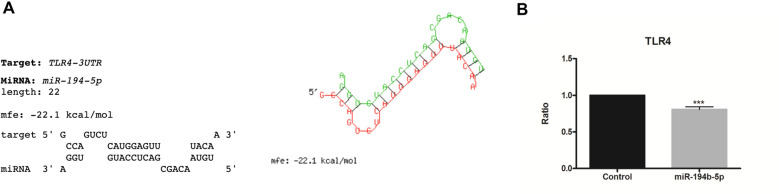
**Prediction analysis of miRNA-target interaction and results of luciferase assay.** **A** Prediction of target sequence in miR-194-5p/TLR4. The highest score obtained from RNAhybrid prediction is showed. **B** Firefly luciferase activity was measured and normalized by the Renila luciferase activity. Data are represented as mean ratio ± SEM from four independent transfection experiments. Two tailed Student’s *t*-test was used to compare samples and significance was set at *P* < 0.05. Asterisk means ***p < 0.001.

Based on the effect of miR-194-5p mimic transfection on gene expression of IPEC-J2 cells infected with *S*. Typhimurium, we selected *TLR4* to test the miRNA-mRNA target interaction using the luciferase reporter assay. Besides previous gene expression data, selection of this miRNA was supported by its biological implication during *S.* Typhimurium infection: TLR4 is the main pathogen recognition receptor, and it triggers the inflammatory signaling cascades in response to *Salmonella* infection [3, 31]. Also, some authors have suggested miR-194 as a regulator of the TLR4 signaling pathway in obesity-driven inflammatory response and necrotizing enterocolitis [28, 32]. As shown in Figure 5, luciferase activity for *TLR4* decreased 24% when miR-194-5p mimic was transfected, validating our bioinformatic predictions of the interaction between the sequences.

**Figure 6:**
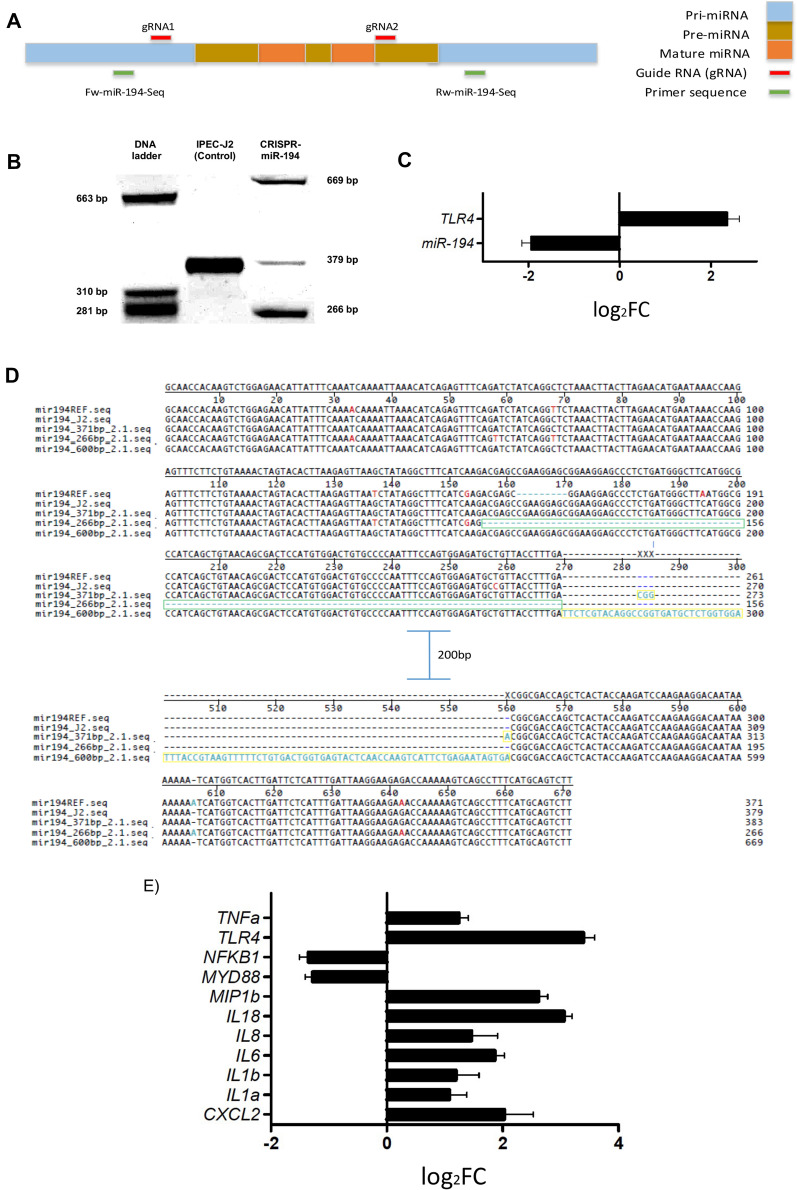
**Inhibition of miR-194 via CRISPR and its effect on inflammatory response.** **A** CRISPR/Cas9 strategy for targeting miR-194. Two gRNA regions are shown at the top panel. miR-194 gRNA was cloned into the vector as detailed in the materials and methods section. **B** DNA cleavage by CRISPR/Cas9 was detected by PCR. **C** Expression levels of miR-194 and its target gene *TLR4* of CRISPR-miR194 clones compared to IPEC-J2 cells control. **D** DNA sequencing confirmed deletions (green boxes) and insertions (yellow boxes) generated by CRISPR/cas9 in miR-194 sequence. **E** Overexpression of *TLR4* and downstream genes in infected CRISPR-miR-194 clones compared to infected IPEC-J2 cells with *S*. Typhimurium.

Additionally, we investigated if the downregulation of miR-194-5p via CRISPR/Cas9 deletion had an effect on *TLR4* and downstream gene expression. To test whether CRISPR/Cas 9 vector efficiently disrupted miRNA function, we transfected IPEC-J2 cells with two different miR-194 guide RNA sequences (gRNA1 and gRNA2), which were located in the pri-miR-194 and pre-miR-194 sequences, respectively (Figure 6A). Following puromycin selection, we characterized the obtained clones, finding that, compared to control cells, CRISPR-miR-194 gRNA vectors induced modifications in the wild-type sequence (Figure 6B). Decreased expression of miR-194 and subsequent *TLR4* overexpression were confirmed in the CRISPR-miR-194 clones (compared to non-edited controls) by qPCR (Figure 6C). Additionally, Sanger sequencing confirmed CRISPR-miR-194 induced deletions and insertions (Figure 6D). Thus, our data demonstrated that CRISPR/Cas9 system is highly effective in abrogating miR-194 regulation on *TLR4* expression by introducing mutations in the pre-miRNA and pri-miRNA sequences in porcine epithelial cells.

To further investigate the function of miR-194 downregulation in the inflammatory response, we determined the expression of the pro-inflammatory genes that were tested for miR-194 mimic transfection in CRISPR-miR-194 clones and IPEC-J2 cells infected with *S*. Typhimurium. We found that compared with IPEC-J2 infected control cells, infected CRISPR-miR-194 clones had increased expression of *TLR4* and downstream genes *(CXCL2*, *IL1α*, *IL1β*, *IL6*, *IL8*, *IL18*, *MIP1b* and *TNFα*). *MYD88* and *NFκB1* were found downregulated in this comparison (Figure 6E). These findings support that either up or downregulation of miR-194 altered the gene expression of *TLR4* and subsequent TLR4 signaling pathway during *S*. Typhimurium infection.

### Determination of the effect of miRNA expression on *S*. Typhimurium invasiveness in IPEC-J2 cells

**Figure 7:**
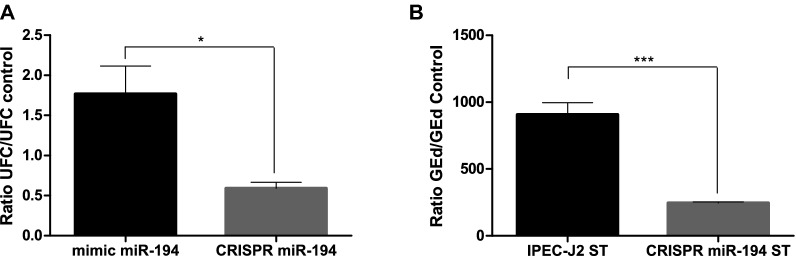
**Effect of miR-194 expression on** ***S.*** **Typhimurium invasion in IPEC-J2 cells.** **A** For gentamicin resistance assay, data are represented as ratio between UFC in mimic transfected or CRISPR-edited cells and UFC in infected cells (means ± SEM). **B** Quantification of *S*. Typhimurium by TaqMan qPCR assay. Data are shown as ratio between the number of *S*. Typhimurium genome equivalents (GEd) in infected IPEC-J2 or CRISPR-edited cells and control cells (means ± SEM). Student’s *t*-test was used to compare controls with infected cells. Asterisk means *P < 0.05; **P < 0.01; ***P < 0.001.

The level of the interaction between *S*. Typhimurium and mimic-transfected/CRISPR-edited IPEC-J2 cells was evaluated by gentamicin resistance assay and TaqMan qPCR. We found that over-expression of miR-194b increases invasion and adhesion of the bacteria (*P* < 0.05), and cells lacking miR-194b (CRISPR-edited) showed a decreased invasion of bacteria (*P* < 0.05) compared to infected control cells (Figure 7). Additionally, we quantified intracellular *S*. Typhimurium DNA in infected IPEC-J2 and CRISPR-miR-194 edited cells, which confirmed the decrease of intracellular *S*. Typhimurium in edited cells. Altogether, invasion assays confirmed the regulatory role of miR-194b in TLR4-mediated *Salmonella* recognition and invasion.

## Discussion

miRNAs are key post-transcriptional regulators in a wide variety of biological processes, including cell proliferation, differentiation, apoptosis, metabolism, immunity, and cancer [33]. Host–pathogen interactions are complicated processes regulated by multiple factors, and miRNAs appear to be important players affecting inflammation and immune response regulation [7]. Many studies have indicated that Gram-negative bacterial infection may trigger complex multisystem responses in the host, and detailed analysis of the pathological process may shed light on the detection of infection in the early stages, allowing evaluation and development of therapies [6]. In this context, regulation of miRNA expression during *Salmonella* infection is emerging as a crucial part of the host response to infection. Several miRNAs have been reported to play a role in *S*. Typhimurium infection in pigs. For example, let-7i-3p is downregulated in porcine ileum, and has been shown to control *Salmonella* adhesion and intracellular replication [8]; also, miR-15a-5p, miR-15b-5p, miR-22, miR-16-5p, miR-421, miR-744 and let-7i-5p (E2F1-dependent miRNAs) were downregulated in *S*. Typhimurium infected porcine ileum and colon, promoting bacterial replication [9].

In this study, ileum samples were used to generate miRNA expression profiles by high throughput sequencing technology. Our study reports that miRNAs such as miR-21, miR-192, miR-143, miR-200 family, and miR-194 are highly abundant in porcine ileum, agreeing with previous reports in the same tissue in other mammals [27, 34–36]. Twenty-eight miRNAs were found differentially expressed following *S*. Typhimurium infection at 2 dpi. The most over-expressed miRNAs in this study were miR-146a/b and miR-223, and such over-expression has been previously associated with the innate immunity regulation and inflammatory response [37], but mechanisms are not clear. Previous studies have reported the induction of miR-146 in macrophages and monocytes in response to microbial infections such as *S*. Typhimurium [38–40]. Increased expression of miR-146a in porcine peripheral blood has been associated with increased fecal shedding counts of the pathogen [41]. Upregulation of this miRNA depends on NFκB [39, 42], producing a negative feedback control of TLR-TRAF6-IRAK1 signaling, which protects against excessive inflammation [43, 44]. miR-146 knockout mice mount an exaggerated inflammatory response to injected LPS, when compared to non-treated animals [45]. miR-223 was significantly over-expressed in ileum at 2 dpi, which also occurs in neutrophils infiltrated in the infected mucosa [46, 47]. Although the relevance of miR-223 in pathological infections and the inflammatory response has been described previously, there is limited information regarding its role in *S*. Typhimurium infection. Dysregulations of miR-223 expression have been observed in many inflammatory disorders such as rheumatoid arthritis, inflammatory bowel disease, osteoarthritis and Crohn’s disease, among others [48], where tissue often undergoes excessive inflammation.

miRNAs such as miR-192/215, miR-194, and the miR-200 family (miR-200a, miR-200b, miR-200c and miR-141 were found downregulated. This group of miRNAs that share a consensus seed sequence has been described in *Gallus gallus* intestinal mucosal layer afflicted with necrotic enteritis [49]. Additionally, these downregulated miRNAs in the current study have also been implicated in the host response to microbial pathogens such as *Listeria monocytogenes* or *Helicobacter pylori* [50–52] suggesting that similar responses to Gram-negative bacterial infection and inflammatory processes could be regulated by miRNAs. Downregulation of miRNAs after *S.* Typhimurium infection in ileum at 2 dpi play a role in epithelial cell proliferation [35, 53, 54], as some studies describe that the expression of miR-192, miR-194, miR-215 and the miR-200 family is necessary for maintaining the epithelial intestinal barrier [55, 56]. In a previous study we demonstrated that *S.* Typhimurium induces the disruption of the epithelial layer during infection at 2 dpi with a complete loss of microvilli [4], which is in agreement with the downregulation of miRNAs involved in maintaining the epithelial intestinal barrier. However, it has been shown that expression of some of these miRNAs such as miR-192/215 can vary depending on the model system used, as it has been reported upregulation of them in *S.* Typhimurium infected human intestinal organoids [57].

In addition to a disruption of the epithelial barrier, a study of gene expression allowed us to determine the existence of a strong inflammatory response in the porcine ileum at 2 dpi [4]. The downregulation of the miR-194 has been associated with inflammatory response [58, 59], which agrees with our results of miR-194 repression and the subsequent transcriptional regulation of specific target genes that control inflammatory processes. A decreased expression of common inflammatory markers such as *IL1α*, *CXCL2* and *TLR4* was observed in infected IPEC-J2 cells when miR-194-5p was over-expressed. This suggests that miR-194 has a direct effect on the inflammatory response. However, Tian et al. found that miR-194 inhibited the TLR4 pathway through targeting a key signal molecule *TRAF6*, which mediates *NFκB* activation and consequently the induction of pro-inflammatory cytokines [28]. Supporting this, Bao et al. showed that miR-194 has an indirect effect on *NFκB* through its target genes *TRIM23* and *C21ORF91*, which are involved in the *NFκB* induction [60]. In addition to the regulation of miR-194 on the TLR4 pathway through TRAF6 and NFκB, we found that miR-194 had *TLR4* as a non-conserved target. This prediction was confirmed using luciferase reporter assay, suggesting that the inflammatory regulation exerted by this miRNA occurs along the TLR4 signaling pathway. To confirm this, we used the CRISPR/Cas9 system to knockdown miR-194a-5p expression. Although miR-194 is derived from two separate loci in the pig genome (Chromosome 10, intron 2 and 12, miRBase), we were able to decrease miR-194a-5p expression, providing a powerful approach for disrupting miRNA sequences and studying the effect on target and downstream genes. The downregulation of miR-194a-5p in IPEC-J2 cells led to an overexpression of *TLR4* in non-infected conditions, with subsequent increase of expression of inflammatory markers such as *CXCL2*, *IL1α*, *IL1β*, *IL6*, *IL8*, *IL18*, *MIP1b* and *TNFα* in IPEC-J2 cells infected with *S*. Typhimurium. Furthermore, we detected lower *S*. Typhimurium invasion when miR-194a-5p expression was decreased by CRISPR/Cas9 system, leading to the overexpression of TLR4. This finding agrees with Arpaia et al., who demonstrated that mice deficient in TLR4 were highly susceptible to the invasion of *S*. Typhimurium [31] supporting the effect of miR-194 on invasiveness and inflammatory response via TLR4.

In summary, our study is a comprehensive analysis of the miRNA expression profile in porcine ileum after *S*. Typhimurium infection. By using integrated analysis, we have identified target genes of the DE miRNAs, which have been validated using luciferase assays, miRNA mimics, and the CRISPR/Cas9 system. The effect of miRNAs in the regulation of cytokines and bacterial infection were investigated, especially in the regulation of miR-194a-5p on the TLR4 pathway. This study will most likely provide new insights into the contribution of the intestinal infection to understand the function of host transcriptional and post-transcriptional landscape during *S*. Typhimurium infection.

## Supplementary Information

**Additional file 1.** **Primers used in this study.** Information related to primers used for miRNA and target gene expression, CRIPSR-Cas9 and luciferase assays.

**Additional file 2.** **Mature miRNA percentage of sequencing read counts in infected and control porcine ileum samples.** File containing the percentage of read counts for each miRNA detected in ileum from *S*. Typhimurium infected and control pigs.

**Additional file 3.** **Differentially expressed miRNAs after** ***S.*** **Typhimurium infection in ileum at 2 dpi.** Differentially expressed miRNAs in porcine ileum, including expression fold change, *p*-values and FDR corrected *p*-values.

**Additional file 4.** **miRNA target gene list generated from differentially expressed miRNA and genes in porcine ileum after** ***S.*** **Typhimurium infection at 2 dpi.** miRNA targets were selected using the miRNA target database miRTarbase (release 6.0) and TargetScan (release 7.0).

**Additional file 5.** **Predicted functions altered by miRNA dysregulation in porcine ileum.** Gene ontology analysis of predicted miRNA target genes that were differentially expressed in porcine ileum after *S*. Typhimurium infection at 2 dpi.

## Data Availability

All sequences were deposited at NCBI Sequence Read Archive (SRA) with accession number PRJNA510944.

## Abbreviations

c.f.u: colony forming units
CHO: Hamster ovary cells
DE: differentially expressed
FC: fold change
FDR: false discovery rate
IPEC-J2 cells: intestinal porcine enterocytes cell line
IRB Barcelona: Institute for Research in Biomedicine in Barcelona
LPS: lipopolysaccharide
MREs: miRNA recognition elements
miRNA: microRNA
PAMPs: pathogen associated molecular patterns
PRRs: pattern-recognition receptors
qPCR: quantitative real-time *PCR*
TLR: Toll-like receptor
small RNA-seq: small RNA sequencing
SRA: Sequence Read Archive
TSA medium: Trypticase soy agar medium
3′UTR: 3′ Untranslated region

## References

[1] European Food Safety Authority, European Centre for Disease Prevention and Control (2021). The European Union one health 2019 zoonoses report. EFSA J.

[2] Broz P, Ohlson MB, Monack DM (2012). Innate immune response to *Salmonella* typhimurium, a model enteric pathogen. Gut Microbes.

[3] Vaure C, Liu Y (2014). A comparative review of toll-like receptor 4 expression and functionality in different animal species. Front Immunol.

[4] Uribe JH, Collado-Romero M, Zaldivar-Lopez S, Arce C, Bautista R, Carvajal A, Cirera S, Claros MG, Garrido JJ (2016). Transcriptional analysis of porcine intestinal mucosa infected with *Salmonella* Typhimurium revealed a massive inflammatory response and disruption of bile acid absorption in ileum. Vet Res.

[5] Jonas S, Izaurralde E (2015). Towards a molecular understanding of microRNA-mediated gene silencing. Nat Rev Genet.

[6] Zhou X, Li X, Wu M (2018). miRNAs reshape immunity and inflammatory responses in bacterial infection. Signal Transduct Target Ther.

[7] Aguilar C, Mano M, Eulalio A (2019). MicroRNAs at the host-bacteria interface: host defense or bacterial offense. Trends Microbiol.

[8] Aguilar C, Cruz AR, Rodrigues Lopes I, Maudet C, Sunkavalli U, Silva RJ, Sharan M, Lisowski C, Zaldivar-Lopez S, Garrido JJ, Giacca M, Mano M, Eulalio A (2020). Functional screenings reveal different requirements for host microRNAs in *Salmonella* and *Shigella* infection. Nat Microbiol.

[9] Aguilar C, Costa S, Maudet C, Vivek-Ananth R, Zaldívar-López S, Garrido J, Samal A, Mano M, Eulalio A (2021). Reprogramming of microRNA expression via E_2_F_1_ downregulation promotes *Salmonella* infection both in infected and bystander cells. Nat Commun.

[10] Collado-Romero M, Martins RP, Arce C, Moreno Á, Lucena C, Carvajal A, Garrido JJ (2012). An in vivo proteomic study of the interaction between *Salmonella* Typhimurium and porcine ileum mucosa. J Proteom.

[11] Hsu SD, Lin FM, Wu WY, Liang C, Huang WC, Chan WL, Tsai WT, Chen GZ, Lee CJ, Chiu CM, Chien CH, Wu MC, Huang CY, Tsou AP, Huang HD (2011). miRTarBase: a database curates experimentally validated microRNA-target interactions. Nucleic Acids Res.

[12] Falgueras J, Lara AJ, Fernández-Pozo N, Cantón FR, Pérez-Trabado G, Claros MG (2010). SeqTrim: a high-throughput pipeline for pre-processing any type of sequence read. BMC Bioinform.

[13] Sun Z, Evans J, Bhagwate A, Middha S, Bockol M, Yan H, Kocher JP (2014). CAP-miRSeq: a comprehensive analysis pipeline for microRNA sequencing data. BMC Genom.

[14] Langmead B (2010). Aligning short sequencing reads with Bowtie. Curr Protoc Bioinform.

[15] Friedländer MR, Chen W, Adamidi C, Maaskola J, Einspanier R, Knespel S, Rajewsky N (2008). Discovering microRNAs from deep sequencing data using miRDeep. Nat Biotechnol.

[16] Robinson MD, McCarthy DJ, Smyth GK (2010). edgeR: a bioconductor package for differential expression analysis of digital gene expression data. Bioinformatics.

[17] Herrera-Uribe J, Zaldivar-Lopez S, Aguilar C, Luque C, Bautista R, Carvajal A, Claros MG, Garrido JJ (2018). Regulatory role of microRNA in mesenteric lymph nodes after *Salmonella* Typhimurium infection. Vet Res.

[18] Mentzel C, Skovgaard K, Córdoba S, Herrera Uribe J, Busk P, Cirera S (2014). Wet-lab tested microRNA assays for qPCR studies with SYBR^®^ Green and DNA primers in pig tissues. Microrna.

[19] Timoneda O, Balcells I, Córdoba S, Castelló A, Sánchez A (2012). Determination of reference microRNAs for relative quantification in porcine tissues. PLoS One.

[20] Livak KJ, Schmittgen TD (2001). Analysis of relative gene expression data using real-time quantitative PCR and the 2(-Delta Delta C(T)) Method. Methods.

[21] Agarwal V, Bell GW, Nam JW, Bartel DP (2015). Predicting effective microRNA target sites in mammalian mRNAs. Elife.

[22] Mlecnik B, Galon J, Bindea G (2019). Automated exploration of gene ontology term and pathway networks with ClueGO-REST. Bioinformatics.

[23] Shannon P, Markiel A, Ozier O, Baliga NS, Wang JT, Ramage D, Amin N, Schwikowski B, Ideker T (2003). Cytoscape: a software environment for integrated models of biomolecular interaction networks. Genome Res.

[24] Krüger J, Rehmsmeier M (2006). RNAhybrid: microRNA target prediction easy, fast and flexible. Nucleic Acids Res.

[25] MIT CRISPR design tool. http://crispr.mit.edu/.

[26] Martins RP, Collado-Romero M, Arce C, Lucena C, Carvajal A, Garrido JJ (2013). Exploring the immune response of porcine mesenteric lymph nodes to *Salmonella enterica* serovar Typhimurium: an analysis of transcriptional changes, morphological alterations and pathogen burden. Comp Immunol Microbiol Infect Dis.

[27] Sharbati S, Friedländer MR, Sharbati J, Hoeke L, Chen W, Keller A, Stähler PF, Rajewsky N, Einspanier R (2010). Deciphering the porcine intestinal microRNA transcriptome. BMC Genom.

[28] Tian H, Liu C, Zou X, Wu W, Zhang C, Yuan D (2015). MiRNA-194 regulates palmitic acid-induced toll-like receptor 4 inflammatory responses in THP-1 cells. Nutrients.

[29] Wang M, Li Z, Zuo Q (2020). miR-194-5p inhibits LPS-induced astrocytes activation by directly targeting neurexophilin 1. Mol Cell Biochem.

[30] Herrera-Uribe J, Liu H, Byrne KA, Bond ZF, Loving CL, Tuggle CK (2020). Changes in H_3_K_27_ac at gene regulatory regions in porcine alveolar macrophages following LPS or PolyIC exposure. Front Genet.

[31] Arpaia N, Godec J, Lau L, Sivick KE, McLaughlin LM, Jones MB, Dracheva T, Peterson SN, Monack DM, Barton GM (2011). TLR signaling is required for *Salmonella* typhimurium virulence. Cell.

[32] Ng PC, Chan KY, Leung KT, Tam YH, Ma TP, Lam HS, Cheung HM, Lee KH, To KF, Li K (2015). Comparative MiRNA expressional profiles and molecular networks in human small bowel tissues of necrotizing enterocolitis and spontaneous intestinal perforation. PLoS One.

[33] Bushati N, Cohen SM (2007). microRNA functions. Annu Rev Cell Dev Biol.

[34] Wu F, Zhang S, Dassopoulos T, Harris ML, Bayless TM, Meltzer SJ, Brant SR, Kwon JH (2010). Identification of microRNAs associated with ileal and colonic Crohn’s disease. Inflamm Bowel Dis.

[35] Liang G, Malmuthuge N, McFadden TB, Bao H, Griebel PJ, Stothard PlL, Guan (2014). Potential regulatory role of microRNAs in the development of bovine gastrointestinal tract during early life. PLoS One.

[36] Liang G, Malmuthuge N, Guan Y, Ren Y, Griebel PJ, Guan LL (2016). Altered microRNA expression and pre-mRNA splicing events reveal new mechanisms associated with early stage *Mycobacterium avium* subspecies *paratuberculosis* infection. Sci Rep.

[37] Staedel C, Darfeuille F (2013). MicroRNAs and bacterial infection. Cell Microbiol.

[38] Monk CE, Hutvagner G, Arthur JS (2010). Regulation of miRNA transcription in macrophages in response to *Candida albicans*. PLoS One.

[39] Taganov KD, Boldin MP, Chang KJ, Baltimore D (2006). NF-kappaB-dependent induction of microRNA miR-146, an inhibitor targeted to signaling proteins of innate immune responses. Proc Natl Acad Sci USA.

[40] Ordas A, Kanwal Z, Lindenberg V, Rougeot J, Mink M, Spaink HP, Meijer AH (2013). MicroRNA-146 function in the innate immune transcriptome response of zebrafish embryos to *Salmonella* typhimurium infection. BMC Genomics.

[41] Huang T, Huang X, Chen W, Yin J, Shi B, Wang F, Feng W, Yao M (2019). MicroRNA responses associated with *Salmonella enterica* serovar typhimurium challenge in peripheral blood: effects of miR-146a and IFN-γ in regulation of fecal bacteria shedding counts in pig. BMC Vet Res.

[42] Quinn SR, O’Neill LA (2011). A trio of microRNAs that control Toll-like receptor signalling. Int Immunol.

[43] Meisgen F, Xu Landén N, Wang A, Réthi B, Bouez C, Zuccolo M, Gueniche A, Ståhle M, Sonkoly E, Breton L, Pivarcsi A (2014). MiR-146a negatively regulates TLR2-induced inflammatory responses in keratinocytes. J Invest Dermatol.

[44] Park H, Huang X, Lu C, Cairo MS, Zhou X (2015). MicroRNA-146a and microRNA-146b regulate human dendritic cell apoptosis and cytokine production by targeting TRAF6 and IRAK1 proteins. J Biol Chem.

[45] Boldin MP, Taganov KD, Rao DS, Yang L, Zhao JL, Kalwani M, Garcia-Flores Y, Luong M, Devrekanli A, Xu J, Sun G, Tay J, Linsley PS, Baltimore D (2011). miR-146a is a significant brake on autoimmunity, myeloproliferation, and cancer in mice. J Exp Med.

[46] Szabady RL, McCormick BA (2013). Control of neutrophil inflammation at mucosal surfaces by secreted epithelial products. Front Immunol.

[47] Tyrkalska SD, Candel S, Angosto D, Gómez-Abellán V, Martín-Sánchez F, García-Moreno D, Zapata-Pérez R, Sánchez-Ferrer Á, Sepulcre MP, Pelegrín P, Mulero V (2016). Neutrophils mediate *Salmonella* Typhimurium clearance through the GBP4 inflammasome-dependent production of prostaglandins. Nat Commun.

[48] Haneklaus M, Gerlic M, O’Neill LA, Masters SL (2013). miR-223: infection, inflammation and cancer. J Intern Med.

[49] Pham TT, Ban J, Hong Y, Lee J, Vu TH, Truong AD, Lillehoj HS, Hong YH (2020). MicroRNA gga-miR-200a-3p modulates immune response via MAPK signaling pathway in chicken afflicted with necrotic enteritis. Vet Res.

[50] Maudet C, Mano M, Eulalio A (2014). MicroRNAs in the interaction between host and bacterial pathogens. FEBS Lett.

[51] Cadamuro AC, Rossi AF, Maniezzo NM, Silva AE (2014). *Helicobacter pylori* infection: host immune response, implications on gene expression and microRNAs. World J Gastroenterol.

[52] Baud J, Varon C, Chabas S, Chambonnier L, Darfeuille F, Staedel C (2013). *Helicobacter pylori* initiates a mesenchymal transition through ZEB1 in gastric epithelial cells. PLoS One.

[53] Pichiorri F, Suh SS, Rocci A, De Luca L, Taccioli C, Santhanam R, Zhou W, Benson DM, Hofmainster C, Alder H, Garofalo M, Di Leva G, Volinia S, Lin HJ, Perrotti D, Kuehl M, Aqeilan RI, Palumbo A, Croce CM (2016). Downregulation of p53-inducible microRNAs 192, 194, and 215 impairs the p53/MDM2 autoregulatory loop in multiple myeloma development. Cancer Cell.

[54] Gaulke CA, Porter M, Han YH, Sankaran-Walters S, Grishina I, George MD, Dang AT, Ding SW, Jiang G, Korf I, Dandekar S (2014). Intestinal epithelial barrier disruption through altered mucosal microRNA expression in human immunodeficiency virus and simian immunodeficiency virus infections. J Virol.

[55] McKenna LB, Schug J, Vourekas A, McKenna JB, Bramswig NC, Friedman JR, Kaestner KH (2010). MicroRNAs control intestinal epithelial differentiation, architecture, and barrier function. Gastroenterology.

[56] Cichon C, Sabharwal H, Rüter C, Schmidt MA (2014). MicroRNAs regulate tight junction proteins and modulate epithelial/endothelial barrier functions. Tissue Barriers.

[57] Lawrence A, Abuaita B, Berger R, Hill D, Huang S, Yadagiri V, Bons B, Fields C, Wobus C, Spence J, Young V, O’Riordan M (2021). *Salmonella enterica* Serovar Typhimurium SPI-1 and SPI-2 shape the global transcriptional landscape in a human intestinal organoid model system. mBio.

[58] Chen Z, Han Y, Deng C, Chen W, Jin L, Chen H, Wang K, Shen H, Qian L (2019). Inflammation-dependent downregulation of miR-194-5p contributes to human intervertebral disc degeneration by targeting CUL4A and CUL4B. J Cell Physiol.

[59] Zhang X, Chen Q, Shen J, Wang L, Cai Y, Zhu KR (2020). miR-194 relieve neuropathic pain and prevent neuroinflammation via targeting FOXA1. J Cell Biochem.

[60] Bao C, Li Y, Huan L, Zhang Y, Zhao F, Wang Q, Liang L, Ding J, Liu L, Chen T, Li J, Yao M, Huang S, He X (2015). NF-κB signaling relieves negative regulation by miR-194 in hepatocellular carcinoma by suppressing the transcription factor HNF-1α. Sci Signal.

